# Mechanism and Context: Making Sense of Adverse Events With GLP-1-based Therapy

**DOI:** 10.1210/jcemcr/luaf232

**Published:** 2025-12-03

**Authors:** Per Gustav Hagelqvist, Tina Vilsbøll, Christine Rode Schwarz

**Affiliations:** Steno Diabetes Center Copenhagen, University of Copenhagen, Herlev 2730, Denmark; Steno Diabetes Center Copenhagen, University of Copenhagen, Herlev 2730, Denmark; Department of Clinical Medicine, Faculty of Health and Medical Sciences, University of Copenhagen, Copenhagen 2200, Denmark; Steno Diabetes Center Copenhagen, University of Copenhagen, Herlev 2730, Denmark

**Keywords:** adverse events, diabetes, GLP-1-based therapy, obesity

Glucagon-like peptide 1 (GLP-1)-based therapies have reshaped the clinical approach to type 2 diabetes and obesity [1]. Long-acting GLP-1 receptor agonists and the glucose-dependent insulinotropic polypeptide/GLP-1 dual-agonist tirzepatide improve glycemic control, reduce body weight, and offer cardiovascular benefits in selected populations [1]. GLP-1 pharmacology includes a glucose-dependent enhancement of insulin secretion, suppression of glucagon in the postprandial state, delayed gastric emptying, and central satiety signaling, which contributes to both benefit and risk. Common adverse effects such as nausea, early satiety, vomiting, and diarrhea are largely dose-related and generally lessen with gradual titration or dose reduction [1]. Less common events, including gallbladder disease and pancreatitis, occur infrequently in trials yet warrant ongoing clinical vigilance [1]. Safe prescribing requires familiarity with incretin pharmacology and individualized clinical judgment regarding initiation, dosing, titration, and monitoring, with explicit attention to patient context. From a pharmacologic and mechanistic perspective, GLP-1-based agents have prolonged elimination half-lives with sustained receptor engagement. Gastric emptying slows and appetite diminishes, and daily energy intake typically falls during dose escalation, particularly early in treatment. In susceptible settings, such as deliberate very low-carbohydrate intake, intercurrent illness with dehydration, or concomitant sodium-glucose cotransporter 2 (SGLT2) inhibitor therapy, these changes may promote a fasting or starvation physiology. This state is characterized by lower endogenous insulin and relative counterregulatory hormone predominance, shifting substrate use toward lipolysis and starvation ketosis; in some individuals, this progression occurs despite near-normal glycemia and presents as euglycemic ketoacidosis (EKA). For women of reproductive potential, clinicians should confirm pregnancy intentions and effective contraception before initiation, counsel that GLP-1 receptor agonists are contraindicated in pregnancy, and discontinue therapy immediately if pregnancy is recognized.

The report by Okeke and colleagues is notable for both initiation and dose [2]. A GLP-1–naïve 34-year-old woman administered semaglutide 2 mg obtained from an online clinic at 7 weeks of gestation. She subsequently developed refractory nausea and vomiting, necessitating intravenous fluids and nutrition support. Symptom onset and resolution closely followed semaglutide's pharmacokinetic profile, with the emesis subsiding 6 days after administration, supporting a direct causal relationship. Early pregnancy physiology already predisposes to nausea through hormonal and autonomic mechanisms; superimposed slowing of gastric emptying and enhanced satiety signaling would be expected to intensify symptoms. Besides the initiating error in a pregnant woman, the starting dose was excessive, far above the labeled initiation at 0.25 mg with gradual escalation. Notably, standard antiemetics and a single dose of corticosteroid provided no relief, arguing that the emesis was driven by ongoing GLP-1 receptor activation after an excessive initiation dose, rather than by dopaminergic or serotonergic pathways. Management should emphasize supportive care with fluids, thiamine, and carbohydrate, allowing exposure to decline, rather than repeated antiemetic escalation.

The case described by Sood and colleagues illustrates the importance of individualized monitoring [3]. A woman without diabetes who had been using semaglutide for weight loss for 7 months presented with 4 days of intractable vomiting, right-upper-quadrant pain, a single episode of hematemesis, and biochemical evidence of metabolic acidosis with markedly elevated ketones, despite near-normoglycemia, consistent with EKA. Endoscopy showed mild esophagitis and gastritis; imaging revealed hepatic steatosis. She improved promptly with fluids, electrolyte replacement, proton-pump inhibition, and bicarbonate plus dextrose. After discharge, she was escalated to 1.7 mg semaglutide without recurrence. This clinical course argues against intrinsic drug intolerance. However, the case suggests several vigilance factors: a prior history of alcohol use disorder, recent viral gastroenteritis, a protein-heavy low-carbohydrate diet, and mild pancreatic enzyme elevations. Thus, factors that raise counterregulatory hormones and lower effective insulin action require patient education at the time of prescription and closer follow-up during uptitration and continued treatment. Taken together, semaglutide may have been 1 of several factors contributing to this ketogenic metabolic complication in a somewhat fragile individual.

Similarly, Louwagie et al described a case of EKA in an individual with type 2 diabetes treated with tirzepatide in combination with an SGLT2 inhibitor [4]. The 35-year-old woman exhibited classic features of EKA, including metabolic acidosis with elevated ketones and normal glucose levels. While SGLT2 inhibitors are well established in the literature as a risk factor for EKA [5], it is notable that the episode occurred approximately 5 weeks after tirzepatide was added. This temporal relationship raises the question of whether the event represents SGLT2 inhibitor-associated EKA, a contribution from reduced intake during tirzepatide titration, or a synergistic effect in a susceptible individual. Although details of the clinical history are limited, the use of potent weight-reducing therapy to improve glycemia in an individual with a normal to low body mass index of 20.7 kg/m^2^ warrants reconsideration of treatment goals and sequence.

The 2 case reports by Sood et al and Louwagie et al raise important questions about the potential role of these GLP-1-based therapies in precipitating EKA. Do these agents directly promote ketogenesis by altering the glucagon-to-insulin balance? In the postprandial state, pharmacologic levels of GLP-1 consistently suppress glucagon and support insulin secretion. During fasting and illness, counterregulatory hormones dominate, and any direct proketogenic effect of GLP-1 receptor activation appears small compared with the impact of carbohydrate deficit, dehydration, and stress [6]. Moreover, large trials such as SELECT (semaglutide in obesity without diabetes; n = 17 604) [7] and across the SURPASS program (tirzepatide in type 2 diabetes, n = 7769) [8] did not report EKA, suggesting that if a risk exists, it is very rare and typically requires additional modifiers such as SGLT2 inhibition, very low carbohydrate intake, or sustained vomiting/starvation.

The cases discussed here should not detract from the substantial value of GLP-1-based therapies, which remain widely used. But they remind us that individualizing therapy with disciplined dosing, attention to context, and anticipatory counseling and monitoring are the simplest and most effective tools to minimize avoidable harm while preserving the broad benefits of GLP-1-based therapies.

## Disclosures

P.G. Hagelqvist reports no conflicts of interest. T. Vilsbøll has served on scientific advisory panels; been part of speaker's bureaus; and served as a consultant to and/or received research support from Amgen, Astra-Zeneca, BMS, Boehringer Ingelheim, Eli Lilly, Gilead, GSK, Mass Medicine, Novo Nordisk, Carmot/Roche, Regor, Sanofi, Sun Pharmaceuticals, and Zealand Pharma. C.R. Schwarz has been part of speaker's bureaus, served as a consultant to, and/or received research support from Eli Lilly and Novo Nordisk.

## Data Availability

Data sharing is not applicable to this article as no datasets were generated or analyzed during the current study.

## Abbreviations

GLP-1: glucagon-like peptide 1
SGLT2: sodium-glucose cotransporter 2
EKA: euglycemic ketoacidosis
